# Demographic and clinical variables affecting mid‐ to late‐life trajectories of plasma ceramide and dihydroceramide species

**DOI:** 10.1111/acel.12369

**Published:** 2015-07-20

**Authors:** Michelle M. Mielke, Veera Venkata Ratnam Bandaru, Dingfen Han, Yang An, Susan M. Resnick, Luigi Ferrucci, Norman J. Haughey

**Affiliations:** ^1^ Departments of Health Science Research and NeurologyMayo ClinicRochesterMN55905USA; ^2^ Department of NeurologyJohns Hopkins University School of MedicineBaltimoreMD21224USA; ^3^ PsychiatryJohns Hopkins University School of MedicineBaltimoreMD21224USA; ^4^ Intramural Research ProgramNational Institute on AgingNational Institutes of HealthBaltimoreMD21224USA

**Keywords:** aging, ceramide, dihydroceramide, human, longitudinal, sex differences

## Abstract

It has been increasingly recognized at the basic science level that perturbations in ceramide metabolism are associated with the development and progression of many age‐related diseases. However, the translation of this work to the clinic has lagged behind. Understanding the factors longitudinally associated with plasma ceramides and dihydroceramides (DHCer) at the population level and how these lipid levels change with age, and by sex, is important for the clinical development of future therapeutics and biomarkers focused on ceramide metabolism. We, therefore, examined factors cross‐sectionally and longitudinally associated with plasma concentrations of ceramides and DHCer among Baltimore Longitudinal Study of Aging participants (*n* = 992; 3960 total samples), aged 55 years and older, with plasma at a mean of 4.1 visits (range 2–6). Quantitative analyses were performed on a high‐performance liquid chromatography‐coupled electrospray ionization tandem mass spectrometer. Linear mixed models were used to assess the relationships between plasma ceramide and DHCer species and demographics, diseases, medications, and lifestyle factors. Women had higher plasma concentrations of most ceramide and DHCer species and showed steeper trajectories of age‐related increases compared to men. Ceramides and DHCer were more associated with waist–hip ratio than body mass index. Plasma cholesterol and triglycerides, prediabetes, and diabetes were associated with ceramides and DHCer, but the relationship showed specificity to the acyl chain length and saturation. These results demonstrate the importance of examining the individual species of ceramides and DHCer, and of establishing whether intra‐individual age‐ and sex‐specific changes occur in synchrony to disease onset and progression.

## Introduction

Ceramides, the central molecular species of the sphingolipid pathway, function both as structural lipids and as second messengers for intra‐ and intercellular signaling affecting cellular growth, proliferation, differentiation, senescence, and apoptosis. At low levels, ceramides are important for injury‐induced cytokine production and activating protein phosphatases and kinases, enzymes involved in stress signaling cascades (Hannun, 1996). However, at high levels, ceramides inhibit cell division and induce cellular dysfunction and apoptosis. Thus, the homeostasis of ceramide metabolism may be critical in regulating lifespan and age‐associated diseases (Cutler & Mattson, 2001; Rao *et al*., 2007). Indeed, it has been increasingly recognized, from the cellular to human level, that perturbations in ceramide metabolism are associated with longevity (Yu *et al*., 2012; Gonzalez‐Covarrubias *et al*., 2013; Cutler *et al*., 2014; Huang *et al*., 2014) and the development and progression of many age‐related diseases including cancer (Alberg *et al*., 2013), atherosclerosis (Ichi *et al*., 2006), insulin resistance and diabetes (Holland *et al*., 2007; Holland & Summers, 2008; Boon *et al*., 2013), Alzheimer's disease (Mielke *et al*., 2012), and Parkinson's disease (Mielke *et al*., 2013).

Notably, each sphingolipid species has multiple chain lengths that are regulated by specific biochemical pathways and contribute to distinct cell functions (Hannun & Obeid, 2011). However, the majority of this research has been conducted at the cellular level. With improved understanding of ceramide chain lengths at the cellular level, and of their role in disease mechanisms, there is a need to quantify and translate this knowledge to clinical and population studies. Yet, compared to other lipids (e.g., cholesterol, triglycerides), there are little data on ceramides at the population level and the intra‐ and interindividual changes in levels of specific carbon chain lengths by age, sex, race, and disease status. Previous, carefully validated, studies have utilized small sample sizes of 10–15 individuals aged 20–40 years (Hammad *et al*., 2010; Bui *et al*., 2012) or have used pooled samples (Quehenberger *et al*., 2010). A recent population‐based study performed plasma lipid profiling in over 1000 individuals, median age of 36, enrolled in the San Antonio Family Heart Study (Weir *et al*., 2013). This study showed important cross‐sectional associations between ceramides and dihydroceramides (DHCer) and psychological and demographic factors. However, to further understand the relationship between individual ceramide species and disease risk, it is important to understand intra‐individual changes with age and onset of disease in people over 50 years.

In this study, we quantified, by age and sex, the individual species of plasma ceramides and DHCer in 992 individuals, aged 55 and older, enrolled in the Baltimore Longitudinal Study of Aging (BLSA). Individuals were followed, with serial measures, up to six visits (mean of 4.1 visits, range 2–6) and 38 years, allowing the assessment of both interindividual variation and intra‐individual changes over time in circulating ceramide concentrations. In addition, we assessed factors associated with variation and changes in these levels including demographics, diseases, medications, lifestyle factors, and other blood lipids.

## Results

### Participant characteristics

The present analyses included 366 women and 626 men with at least two blood draws (mean of 4.1, range 2–6) after the age of 55 years who were randomly selected from the BLSA. Participant characteristics at the first blood draw, by sex, are shown in Table 1. Compared to men, women were slightly older (mean: 64.6 vs. 62.7 years, *P *=* *0.001), included a higher proportion of African Americans (18.3% vs. 9.3%, *P *<* *0.001), had fewer years of education (mean: 15.7 vs. 16.8, *P *<* *0.001), lower waist–hip ratio (WHR; mean: 0.8 vs. 0.9, *P *<* *0.001), and higher total cholesterol (mean: 229.8 vs. 221.7, *P *=* *0.003). Women were less likely than men to have ever smoked (27.0% vs. 72.2%, *P *<* *0.001) or to have a diagnosis of prediabetes/diabetes (20.9% vs. 59.2%, *P *<* *0.001) or myocardial infarction (0.3% vs. 3.8%, *P *=* *0.001).

**Table 1:** Participant characteristics, by sex, at the first blood draw with measures of ceramides and dihydroceramides

Variable	Men		Women		P‐value
	Total N	N (%)/mean (SD)	Total N	*N* (%)/mean (SD)	
Age	626	62.7 (7.9)	366	64.6 (8.4)	0.001
Race	626		366		<0.001
Caucasian		568 (90.7%)		299 (81.7%)	
African American		58 (9.3%)		67 (18.3%)	
Education, years	626	16.8 (2.7)	366	15.7 (2.5)	<0.001
Ever smoking	626	452 (72.2%)	366	172 (47.0%)	<0.001
Body mass index	624	26.2 (3.5)	366	25.7 (4.6)	0.062
Waist–hip ratio	486	0.9 (0.1)	355	0.8 (0.1)	<0.001
Hypertension	626	178 (28.4%)	366	84 (23.0%)	0.059
Myocardial infarction	626	24 (3.8%)	366	1 (0.3%)	0.001
Atrial fibrillation	626	6 (1.0%)	366	1 (0.3%)	0.213
Diabetes	626		366		<0.001
None		256 (40.9%)		286 (78.1%)	
Prediabetes		329 (52.6%)		67 (18.3%)	
Diabetes		41 (6.6%)		13 (2.6%)	
Chronic kidney disease	626	12 (1.9%)	366	13 (3.6%)	0.113
Any cancer	626	28 (4.5%)	366	18 (4.9%)	0.748
APOE E4 allele	464	120 (25.9%)	320	94 (29.4%)	0.278
Total cholesterol	595	221.7 (40.9)	351	229.8 (40.9)	0.003
Triglycerides	578	118.3 (80.1)	341	108.8 (81.1)	0.080
Statin use	626	37 (5.9%)	366	27 (7.4%)	0.364

The diagnoses of diabetes and prediabetes at each visit were established by combining information on medications, fasting glucose, and glucose levels at 2 h of a standard glucose tolerance test. In particular, participants who were taking antidiabetes medication or had a fasting glucose > 126 mg dL^−1^ and/or a 2‐h glucose > 200 mg dL^−1^ were defined as diabetics. Among those who had no diabetes, participants with fasting glucose > 100 mg dL^−1^ and/or a 2‐h glucose > 140 mg dL^−1^ were defined as having prediabetes.

### Plasma ceramide and dihydroceramide concentrations by age and sex at the first blood draw

Table 2 provides the cross‐sectional raw means and SD of the individual ceramide and DHCer species at the first blood draw grouped in 10‐year intervals and by sex. The cross‐sectional relationships between the plasma concentrations, by age and sex, differed by ceramide class and species. For example, mean concentrations of ceramide C16:0 were lower in women than in men at ages 55–64, but higher in women at ages 65–74 years. In contrast, concentrations of ceramide C24:0 were cross‐sectionally lower in women than in men over the entire age span tested (55–94). Dihydroceramides are precursors to ceramide, although they also appear to have independent biological functions. Two DHCer species were readily detectable in plasma (C20:0 and C24:0) and cross‐sectionally increased with age in men and women.

**Table 2:** Plasma ceramides and dihydroceramides by age and sex at the first blood draw

Plasma lipids (ng mL^−1^)	Aged 55–64 years			Aged 65–74 years			Aged ≥ 75 years		
	N	Mean (SD)	Range	N	Mean (SD)	Range	N	Mean (SD)	Range
*Ceramides*									
C16:0									
Women	205	97.7 (60.6)c	15.6–339	104	113.7 (65.8)a	15–302	43	111.0 (70.4)	25.9–348
Men	415	122.1 (64.8)	10.8–344	118	109.9 (57.4)	27.7–326	67	120.8 (64.8)	30.9–326
C18:0									
Women	211	74.9 (52.8)a	12.9–307	105	80.3 (45.6)	18.3–282	46	92.8 (54.4)a	16.9–243
Men	419	83.1 (46.7)	1.1–308	124	90.8 (57.9)	13.3–302	69	86.6 (48.8)	18.9–235
C20:0									
Women	212	170.8 (95.1)	21.1–579	105	177.1 (95.5)b	33.5–507	48	212.7 (110.7)c	53.9–606
Men	426	180.1 (90.5)	34.1–625	127	196.0 (96)	25.8–533	69	198.7 (104.7)	34.7–562
C22:0									
Women	210	1898.3 (929)	176–5660	104	1903.1 (763.3)b	587–3840	46	2114.6 (850.4)c	854–4300
Men	426	1968.0 (852.5)	274–5400	126	1931.8 (731.4)	444–3790	69	2130.6 (993.2)	698–5140
C24:0									
Women	209	7537.0 (3735.3)	1330–23 300	104	7588.6 (3780.8)b	1810–22 300	47	8386.8 (3924.1)c	2740–23 200
Men	426	8155.8 (4025.8)	1120–23 700	126	8034.8 (3365.9)	2220–18 400	69	8707.0 (4468.7)	2450–22 000
C26:0									
Women	207	119.0 (72.7)b	19.0–376.0	104	122.4 (82.6)c	23.3–389.0	46	137.1 (83.7)	26.1–339.0
Men	421	116.5 (72.4)	13.9–395.0	126	116.7 (67.4)	21.5–347.0	65	128.8 (81.8)	22.4–393.0
C22:1									
Women	208	23.5 (15.4)c	2.8–78.8	105	24.9 (15.7)c	1.2–80.4	47	27.9 (15.2)c	8.1–81.5
Men	422	21.3 (15.6)	0.9–79.3	126	20.2 (13.1)	1.7–70	68	24.3 (16.3)	2.1–70.5
C24:1									
Women	210	307.2 (237.4)c	8.0–1610.0	105	362.8 (266.4)c	13.0–1220.0	48	394.0 (268.4)c	25.1–1090.0
Men	427	295.5 (247.2)	2.8–1530.0	126	332.2 (288.3)	8.5–1330.0	69	363.3 (278.6)	12.1–1620.0
*DihydroCeramides*									
C20:0									
Women	211	3.2 (1.8)	0.2–11.2	104	3.2 (1.8)a	0.4–8.6	47	3.8 (1.8)c	0.9–9.3
Men	422	3.4 (1.7)	0.1–11.0	126	3.7 (1.8)	0.5–8.7	69	3.8 (1.9)	0.4–11.0
C24:0									
Women	211	29.2 (22.6)b	1.5–117.0	104	32.5 (22.9)c	2.1–87.7	48	30.9 (20.6)c	1–82.3
Men	427	28.7 (22.6)	1.4–125.0	127	32.7 (22.7)	0.8–105.0	69	29.5 (22.9)	0.9–117.0

*T*‐tests used to compare cross‐sectional sex differences within each age group. a, *P* < 0.05; b, *P* < 0.01; c*, P* < 0.001.

### Longitudinal intra‐individual stability

The mean (SD) number of serial plasma samples available for each subject with measured ceramides and metabolites was 4.1 (0.7). We included 3960 total samples from the 992 participants that were collected over an average of 14.3 (6.7) years, with a range of 2.0–38.9 years. Storage time was not associated with ceramide or DHCer concentrations, suggesting that these classes of lipids were stable in long‐term −80 °C storage. The intraclass correlation (ICC) of the various ceramide species, a measure of how well different molecular classes track over time, ranged from 0.14 to 0.22 (all *P *<* *0.0001). The ICC of DHCer was similar and ranged from 0.14 to 0.20 (all *P *<* *0.0001).

### Variables cross‐sectionally and longitudinally associated with ceramide and dihydroceramide concentrations

**Figure 1:**
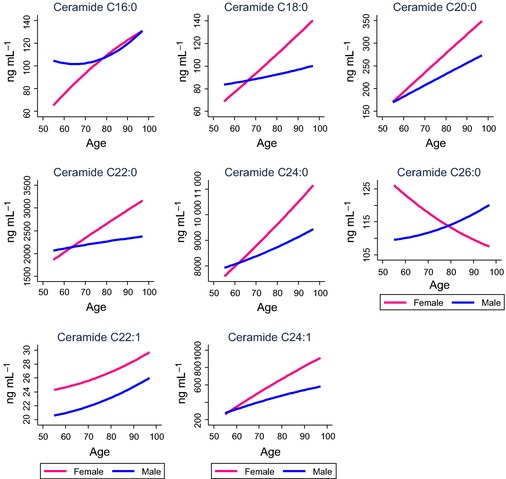
Plasma ceramides by age and sex. Concentrations are based on predicted values obtained in linear mixed models and controlling for additional factors specific to each model (see Table 3).

**Figure 2:**
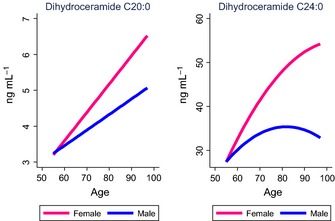
Plasma dihydroceramides by age and sex. Concentrations are based on predicted values obtained in linear mixed models and controlling for additional factors specific to each model (see Table 4).

We next identified factors associated cross‐sectionally and longitudinally, in a time‐dependent manner, with each of the ceramide and DHCer species using linear mixed models. We identified the most parsimonious models and distinguished clinical and demographic variables most strongly associated with each ceramide class and individual ceramide species. Table 3 and Fig. 1 provide the model and model‐fitted predicted values for the ceramides. Table 4 and Fig. 2 provide the model and model‐fitted predicted values for the DHCer.

**Table 3:** Variables cross‐sectionally and longitudinally associated with specific carbon chain lengths of plasma ceramides in the Baltimore Longitudinal Study of Aging

Covariates	C16:0		C18:0		C20:0		C22:0		C24:0		C26:0		C22:1		C24:1	
	Baseline	Time‐dependent	Baseline	Time‐dependent	Baseline	Time‐dependent	Baseline	Time‐dependent	Baseline	Time‐dependent	Baseline	Time‐dependent	Baseline	Time‐dependent	Baseline	Time‐dependent
	*b* (SE)	*b* (SE)	*b* (SE)	*b* (SE)	*b* (SE)	*b* (SE)	*b* (SE)	*b* (SE)	*b* (SE)	*b* (SE)	*b* (SE)	*b* (SE)	*b* (SE)	*b* (SE)	*b* (SE)	*b* (SE)
Age	1.69 (0.41)c	–	1.67 (0.37)c	–	4.24 (0.66)c	–	30.83 (6.56)c	–	81.97 (28.12)b	–	−0.49 (0.20)a	–	0.11 (0.05)a	–	16.02 (1.99)c	–
Age^2^	−0.01 (0.02)	–	0.002 (0.01)	–	−0.001 (0.02)	–	−0.04 (0.15)	–	0.21 (0.67)	–	0.004 (0.01)	–	0.002 (0.002)	–	−0.04 (0.05)	–
Men	8.97 (3.39)b	−1.41 (0.32)c	−4.85 (2.82)	−1.30 (0.27)c	−27.78 (5.32)c	−1.76 (0.38)c	−164.55 (47.85)b	−23.54 (4.78)c	−399.62 (201.41)a	−48.52 (19.57)a	−6.11 (2.47)a	0.69 (0.25)b	−3.70 (0.79)c		−109.83 (15.48)c	−8.19 (1.44)c
African American	3.99 (3.29)	−1.09 (0.37)b	−15.29 (3.04)c	−0.91 (0.34)b	−32.16 (5.91)c	−1.96 (0.66)b	−311.66 (51.87)c	−11.39 (6.15)	−878.05 (214.59)c	−48.64 (25.55)					−87.20 (17.10)c	−5.96 (1.86)b
Education	0.31 (0.40)	0.07 (0.04)	0.45 (0.36)	0.07 (0.04)a	1.21 (0.71)	0.12 (0.07)	9.99 (6.17)	1.18 (0.64)							4.69 (2.04)a	0.38 (0.19)
APOE E4	−0.20 (2.64)	−0.50 (0.27)					−74.45 (41.19)									
BMI	−0.94 (0.27)b		0.95 (0.25)c		1.50 (0.47)b		10.55 (4.26)a		−3.77 (18.07)		−0.31 (0.28)		0.39 (0.07)c		2.64 (1.34)a	
WHR	7.65 (15.24)	4.45 (1.49)b	52.70 (13.61)c	4.47 (1.32)b	119.96 (26.43)c		480.10 (242.08)a	71.72 (23.83)b	1505.79 (1041.71)	181.70 (102.07)			5.02 (4.03)	1.00 (0.32)b	304.96 (75.95)c	22.16 (7.20)b
CKD	18.58 (3.36)c		22.45 (3.13)c		35.84 (6.99)c	1.14 (0.65)	296.15 (56.51)c		796.78 (241.71)b		7.59 (4.27)		3.19 (0.91)c		120.94 (18.00)c	
Statin use			14.29 (2.28)c		21.74 (4.38)c										48.10 (12.89)c	
Pre‐diabetes	4.09 (2.11)		5.29 (1.92)b						−216.21 (146.25)	−18.56 (15.11)			−0.28 (0.56)	−0.05 (0.06)		
Diabetes	−1.52 (3.24)		3.49 (3.01)						6.93 (234.23)	−51.44 (23.23)a			2.28 (0.92)a	−0.16 (0.09)		
Hypertension	−3.81 (2.08)						−55.68 (33.26)									
Smoker			0.27 (2.07)	0.53 (0.20)b									−1.11 (0.59)			
Triglycerides	−0.02 (0.01)	0.004 (0.001)b	0.03 (0.01)b	0.003 (0.001)a	0.14 (0.02)c		2.04 (0.23)c		3.09 (0.96)b				0.01 (0.004)a			
Cholesterol	0.20 (0.03)c		0.11 (0.02)c	−0.004 (0.002)	0.30 (0.05)c		4.18 (0.41)c		23.25 (1.74)c	0.34 (0.17)a	0.39 (0.03)c	0.01 (0.003)	0.03 (0.01)c	0.001 (0.001)	0.54 (0.13)c	

APOE E4, presence of at least one APOE E4 allele; BMI, body mass index; WHR, waist–hip ratio; CKD, chronic kidney disease. We forced age, age 2, sex, and BMI into all models then used backward selection with *P *<* *0.10 to determine which variables to include in the final model of each ceramide species and carbon chain length. a, *P *<* *0.05; b, *P *<* *0.01; c, *P *<* *0.001.

**Table 4:** Variables cross‐sectionally and longitudinally associated with plasma dihydroceramides C20:0 and C24:0 in the Baltimore Longitudinal Study of Aging

Covariates	C20:0		C24:0	
	Baseline	Time‐dependent	Baseline	Time‐dependent
	*b* (SE)	*b* (SE)	*b* (SE)	*b* (SE)
Age	0.08 (0.01)c		0.77 (0.16)c	
Age^2^	0.0001 (0.0003)		−0.01 (0.004)b	
Men	−0.49 (0.10)c	−0.04 (0.007)c	−7.55 (1.23)c	−0.51 (0.11)c
African American	−0.56 (0.11)c	−0.03 (0.01)b	−6.61 (1.30)c	−0.35 (0.15)a
Education	0.02 (0.01)	0.003 (0.001)a	0.50 (0.16)b	
BMI	0.02 (0.01)a		0.11 (0.11)	
WHR	2.26 (0.51)c		14.67 (6.25)a	1.14 (0.59)
CKD	0.74 (0.12)c		9.97 (1.47)c	
Statin use	0.35 (0.08)c		2.07 (1.06)	
Triglycerides	0.002 (0.001)c			
Cholesterol	0.01 (0.001)c		0.09 (0.01)c	

BMI, body mass index; WHR, waist–hip ratio; CKD, chronic kidney disease. We forced age, age^2^, sex, and BMI into all models then used backward selection with *P *<* *0.10 to determine which variables to include in the final model of each ceramide species and carbon chain length. a, *P *<* *0.05; b, *P *<* *0.01; c, *P *<* *0.001.

### Associations with demographic variables

Examining the within‐individual changes using linear mixed models and adjusting for covariates, all ceramide and DHCer species significantly increased with age in both men and women, but women showed a steeper trajectory of increase. The single exception was ceramide C26:0, which decreased in women and increased in men with age (Tables 3 and 4, predicted values are shown in Figs 1 and 2). Compared to Caucasians, African Americans exhibited lower plasma concentrations of most ceramide and DHCer species at the first blood draw, and also had less age‐related increases after controlling for potentially confounding variables. More years of education were associated with greater age‐related increases in ceramides C18:0 and C24:1 and DHCer C20:0.

### Associations of anthropometric measures and diabetes

Both WHR and body mass index (BMI) were associated with higher concentrations of ceramides and DHCer at the first blood draw, but the relationship between baseline WHR and plasma ceramide and DHCer concentrations was stronger. A higher WHR was also associated with a steeper trajectory of age‐related increases in these ceramide species. As WHR is correlated with type II diabetes, we next determined the association of plasma ceramides and DHCer with diabetes. Prediabetes was cross‐sectionally associated with higher ceramide C18:0 (*b* = 5.29, *P *<* *0.01) and diabetes with higher C22:1 (*b* = 2.28, *P *<* *0.05), and this association was maintained longitudinally (i.e., no interaction with time). There was an interaction between diabetes and ceramide C24:0 such that with increasing age, diabetes was associated with lower ceramide concentrations (*b* = −51.44, *P *<* *0.05). There were no cross‐sectional or longitudinal associations between DHCer and prediabetes or diabetes.

### Associations with blood cholesterol, triglycerides, statins, and APOE E4 genotype

Higher plasma concentrations of cholesterol were cross‐sectionally associated with higher concentrations of all ceramide and DHCer species (*P *<* *0.001). However, higher cholesterol was only associated with a steeper age‐related increase in ceramide C24:0 (*b* = 0.34, *P *<* *0.05). Similarly, higher plasma concentrations of triglycerides were cross‐sectionally associated with most ceramide species, with the exceptions of the very long chain ceramides C26:0 and C24:1 and DHCer C24:0. Higher triglycerides were associated with steeper age‐related increases in ceramide C18:0, but not other chain lengths. Unexpectedly, we found that statin use was associated with higher ceramide C18:0, C20:0, and C24:1 and DHCer C20:0 levels at baseline, but the proportion of individuals taking statins at baseline was < 6%. There were no associations for any ceramides or DHCer with APOE E4 genotype.

### Associations with other age‐related diseases

There were essentially no associations between circulating plasma ceramides or DHCer and hypertension, myocardial infarction, or smoking status. Individuals with chronic kidney disease did have higher concentrations of all ceramides, with the exception of C26:0, and DHCer at the first study visit, and these elevated levels were maintained longitudinally (i.e., no interaction with time).

## Discussion

Over the past two decades, the importance of ceramides and ceramide metabolites as mediators, and biomarkers, of complex disease processes has emerged. Indeed, it is increasingly recognized at the cellular level that the homeostasis of ceramide metabolism is critical in regulating lifespan and age‐associated diseases (Cutler & Mattson, 2001; Rao *et al*., 2007; Huang *et al*., 2014). However, given the longer lifespan and greater complexity of sphingolipid metabolism in mammals, fewer studies have translated this cellular work to humans and large‐scale clinical and epidemiological studies of these lipids are lacking. As the biological pathways and consequences of ceramide metabolism continue to be elucidated for specific age‐associated diseases and treatments are developed, a more thorough understanding of the factors that affect ceramides in humans and how they change with age is needed. This information could be useful for identifying biomarkers of disease risk (similar to cholesterol) or for assessing treatment response. Therefore, we sought to understand how age and sex influence plasma ceramide and DHCer concentrations in a longitudinal cohort of 992 individuals. In this initial, descriptive analysis, we examined the longitudinal associations of plasma ceramide and DHCer concentrations with multiple demographic and clinical factors.

The quantification of the individual chain lengths is especially important in understanding the mechanistic pathways influenced by selected molecular species. Sphingolipid metabolic pathways are diverse, dynamic, and exceedingly complex. There are over 28 known enzymes that can act on ceramide as either substrate or product (reviewed in Hannun & Obeid, 2011). Some of these synthetic and catabolic enzymes show specificity for certain carbon chain lengths, but there is considerable redundancy across many of the enzymes. Therefore, in most situations, modifying an enzyme would modify several different sphingolipid species. A recent paper highlighted distinct cellular functions for the different ceramide carbon chain lengths and hydroxylations in yeast (Montefusco *et al*., 2013). Thus, it is not surprising that we also found that the relationship between the plasma ceramides and DHCer and multiple factors varied by carbon chain length. While the measurement of total lipid species (all ceramides, all DHCer) may be easier to measure from a clinical standpoint, much information will be missed by not examining the specific carbon chain lengths.

A number of cross‐sectional studies have reported sex differences in circulating ceramides (Hammad *et al*., 2010; Bui *et al*., 2012; Ishikawa *et al*., 2013; Weir *et al*., 2013). Typically, women have higher blood levels of most ceramides compared with men. In the present study, we also found greater intra‐individual increases in most ceramides with age for women compared to men. The only exception was ceramide C26:0, which decreased with age among women, but increased with age among men. All women would have been postmenopausal at baseline, given they were aged 55 and older, and it is possible that estrogen loss could be contributing to the general ceramide increase with age. However, the reason for the decrease in ceramide C26:0 among women is not known. While there is a dearth of studies examining this relationship, the sphingolipid pathway is a target of estrogens and phytoestrogens (Lucki & Sewer, 2010). These findings warrant future research into the interrelationships between sex hormones and the sphingolipid pathway in moderating sex‐ and age‐specific disease risk.

Ceramide metabolism has repeatedly been found, using both *in vitro* and *in vivo* models, to induce insulin resistance via the phosphatidylinositol‐3‐kinase (PI3K)/Akt pathway (Holland & Summers, 2008). Ceramide blocks the translocation of Akt/protein kinase B (PKB) to the plasma membrane, thus inhibiting insulin signaling (Stratford *et al*., 2001), and promotes the dephosphorylation of Akt/PKB by protein phosphatase 2A (Chavez *et al*., 2003). In rodent models of type 2 diabetes, increases in islet ceramides precede beta‐cell dysfunction (Lee *et al*., 1994). Translating this work to humans, plasma ceramides have been reported to be higher in both patients with prediabetes and patients with diabetes. Elevated levels of ceramides C18:0, C20:0, and C22:0 were reported in adolescents and young adults with type 2 diabetes (Lopez *et al*., 2013). In the present study, individuals with prediabetes had higher levels of ceramide C18:0 and those with diabetes had higher C22:1. Notably, ceramide synthase 1 has specificity for C18 chain lengths and is primarily expressed in skeletal muscles. Ceramide muscle content has been associated with insulin resistance in both normal individuals and athletes, suggesting that elevated ceramides may be a mechanism by which insulin resistance is also associated with accelerated decline in muscle mass and strength (Amati *et al*., 2011; Dube *et al*., 2011; Kalyani *et al*., 2012).

The majority of studies examining sphingolipids and cardiovascular diseases have focused on sphingomyelins or the cardioprotective effects of sphingosine‐1‐phosphate. Plasma ceramides C16:0, C22:0, C24:0, and C24:1 were elevated in both spontaneously hypertensive rats and 19 treatment naïve patients with stage 1–3 hypertension compared to controls (Spijkers *et al*., 2011). In the present study, we did not find an association between any of the ceramides or DHCer and hypertension after adjusting for age, sex, BMI, diabetes, and other covariates. However, virtually all BLSA participants with high blood pressure were treated with antihypertensive medications, and the effect of the different classes of antihypertensives on plasma ceramide levels is not known.

Previous cellular studies have determined that high ceramide levels cause renal damage, for example (Itoh *et al*., 2006), but few have examined specific carbon chain lengths. One study reported higher absolute values of ceramides C16:0, C22:0, and C24:0 in the kidney extracted from CD‐1 mice 2 and 18 h after ischemia–reperfusion injury (Kalhorn & Zager, 1999). Translating to humans, a recent study found higher levels of all serum ceramides, except C18:1, in children with chronic kidney disease (CKD; Mitsnefes *et al*., 2014). Consistently, we also found that individuals with CKD had high levels of several ceramides and DHCer, even after adjusting for diabetes. However, longitudinal research is needed to determine whether blood ceramides are risk factors for CKD or are markers of disease progression and severity.

The levels of plasma ceramides and DHCer did not vary by APOE E4 genotype in this study. Our findings are consistent with a genomewide association study of circulating sphingolipids (Demirkan *et al*., 2012), and with clinical (Han *et al*., 2011) and animal (Sharman *et al*., 2010) studies that have also not found blood ceramide levels to vary by APOE genotype. However, we previously found that levels of CSF very long chain ceramides with chain lengths of C20–C26 were higher in APOE E4 carriers compared with noncarriers (Mielke *et al*., 2014). Further, studies of Alzheimer (Bandaru *et al*., 2009) and HIV dementia brains (Cutler *et al*., 2004) and aortic tissue levels of APOE knockout mice (Kobayashi *et al*., 2013) have found APOE genotype alters ceramide levels in these compartments. Thus, the relationship between ceramides and APOE genotype likely varies depending on the compartment and disease state.

Limitations of the study warrant consideration. First, the BLSA is a community‐dwelling volunteer cohort that is predominantly white, of upper‐middle socioeconomic status, and with an above average educational level. While this may hinder generalizability, the relative homogeneity of the sample may be seen as an asset because the majority of individuals have good access to medical care and have remained relatively healthy over the follow‐up interval. Second, there are multiple ways ceramide can be synthesized and metabolized and these functions are highly compartmentalized. Therefore, for some diseases, tissue‐specific lipid measures (e.g., skeletal muscle for diabetes) may be a better biomarker. Because the collection of blood is noninvasive and more acceptable and feasible for serial measures, we initially focused on the characterization of ceramides and metabolites in this medium. Lastly, ceramides are hydrophobic, so they are carried on lipoproteins in the blood, with the greatest concentrations in VLDL and LDL (Hammad *et al*., 2010) and the ceramide transporter (CERT) (Mencarelli *et al*., 2010). The composition and quantification of the specific acyl chain lengths of ceramides and DHCer on lipoproteins or CERT may differ by age and with disease onset. However, to quantitate all of the lipids by specific lipoproteins and CERT would require many more runs and would take a significantly greater amount of time and effort. Thus, the present work is the first step in beginning to understand the relationship between plasma ceramides and DHCer with age and disease processes.

## Experimental procedures

### Participants

Initiated in 1958, the BLSA is a longitudinal cohort study of community‐dwelling individuals aimed at examining the physiological and psychological aspects of aging (Shock *et al*., 1984). At each study visit, participants underwent an extensive medical examination, neuropsychological battery, blood draw, medical history, and medication review. Historically, BLSA visits occurred every 2 years. In 2003, the sampling times were modified because historical data indicated nonlinear changes at the oldest ages. To improve sampling of the epoch with accelerated physical and cognitive change, individuals aged 80 and older have been evaluated annually since 2003. The protocol was approved by the local Institutional Review Board, and written informed consent was obtained. Since its inception in 1958, over 3100 BLSA participants have contributed data on the aging process. Due to the cost and time constraints of measuring the plasma ceramides, the present analyses included 992 individuals. These individuals were randomly selected from the BLSA participants and had at least two blood draws (mean of 4.1, range 2–6) after the age of 55 years to allow for measurement of within‐individual changes in the ceramide levels starting at mid‐life. The individuals included in the current analyses are representative of the larger BLSA cohort with regard to demographics and health characteristics.

Blood samples were drawn at all visits from the antecubital vein between 7 and 8 AM after an overnight fast (Shock *et al*., 1984). Participants were not allowed to smoke, engage in physical activity, or take medications before the sample was collected. Plasma samples were immediately processed, catalogued, and stored at −80 °C.

### Description of variables examined in relation to lipid levels

All variables were assessed at each visit using the same methods. Demographic variables included age, sex, race, and years of education. Height (in meters) and weight (in kilograms) were measured to calculate BMI. Waist–hip ratio was measured in the standing position using a flexible metal tape. Smoking status was obtained by a questionnaire, and individuals were classified as ever or never smokers. Medical history information included hypertension, myocardial infarction, atrial fibrillation, angina, chronic heart failure, and CKD. The diagnoses of diabetes and prediabetes at each visit were established by combining information on medications, fasting glucose, and glucose levels at 2 h of a standard glucose tolerance test. In particular, participants who were taking antidiabetes medication or had a fasting glucose > 126 mg dL^−1^ and/or a 2‐h glucose > 200 mg dL^−1^ were defined as diabetics. Among those who had no diabetes, participants with fasting glucose > 100 mg dL^−1^ and/or a 2‐h glucose > 140 mg dL^−1^ were defined as having prediabetes.

Plasma total cholesterol and triglycerides were determined by an enzymatic method (Abbott Laboratories ABA‐200 ATC Biochromatic Analyzer, Irving, TX, USA). APOE E4 genotype was determined using polymerase chain reaction amplification of leukocyte deoxyribonucleic acid followed by *Hha*I digestion and product characterization (Hixson & Vernier, 1990) or TaqMan, relying on several single nucleotide polymorphisms around the APOE gene (Koch *et al*., 2002).

### Lipid extraction and LC/ESI/MS/MS analysis

A crude lipid extraction of plasma was conducted using a modified Bligh and Dyer procedure with ceramide C12:0 included as an internal standard (Avanti Polar Lipids, Alabaster, AL, USA) (Bandaru *et al*., 2013). Plasma extracts were dried in a nitrogen evaporator (Organomation Associates Inc., Berlin, MA, USA), and resuspended in pure methanol just prior to analysis. An autosampler (LEAP Technologies Inc., Carrboro, NC, USA) injected extracts into an HPLC (PerkinElmer, Waltham, MA, USA) equipped with a reverse‐phase C18 column (Phenomenex, Torrance, CA, USA). Ceramide species were separated by gradient elution at the flow rate of 400.0 μL min^−1^. The mobile phase A consisted of 85% methanol, 15% H_2_O, and 5 mm ammonium formate. Mobile phase B consisted of 99% methanol, 1% formic acid, and 5 mm ammonium formate. Gradient conditions were as follows: a gradual increase from 100% A to 100% B over 0.5 min, hold at 100% B for 4.5 min, then a decline from 100% to 0% B during the next 1 min.

**Figure 3:**
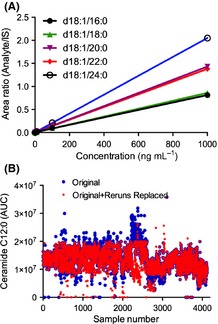
(A) Five‐point standard curves for the indicated ceramide species. (B) Initial and rerun data showing values obtained for the internal standard ceramide C12:0.

**Figure 4:**
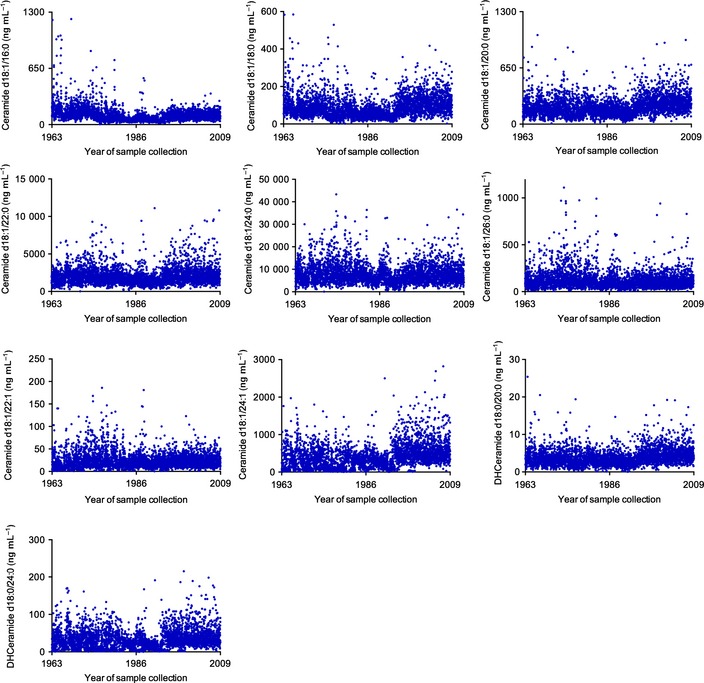
Scatterplots of all plasma ceramide and dihydroceramide species by date of visit show a random distribution, suggesting that these lipids were stable during long‐term storage.

Eluted sample was injected into an electrospray ion source coupled to a triple quadrupole mass spectrometer (API3000; AB Sciex Inc., Thornhill, ON, Canada) (Bandaru *et al*., 2013). Instrument parameters were as follows: ion spray voltage (V) 5500 at a temperature of 80 °C with a nebulizer gas of 8 psi, curtain gas 8 psi, and collision gas 4 psi. The declustering potential was 80 V, focusing potential 400 V, entrance potential 10 V, collision energy 30 V, and collision cell exit potential 18 V. Analysis was conducted by multiple reaction monitoring. MS/MS transitions (*m/z*) of individual ceramide molecular species for sphingomyelin precursor and product ions are provided in Table 5. Eight‐point calibration curves (0.1–1000 ng mL^−1^) were constructed by plotting area under the curve (AUC) for each calibration standard d18:1/C16:0, d18:1/C18:0, d18:1/C20:0, d18:1/C22:0, d18:1/C24:0 normalized to the internal standard. Correlation coefficients (*R* ^2^) obtained were > 0.999 (Fig. 3A). Ceramide concentrations were determined by fitting the identified ceramide species to these standard curves based on acyl chain length. Internal standards were run daily, and AUCs plotted weekly, to track instrument efficiency. Plasma extracts were re‐analyzed if the internal standard deviated more than 25% of the median value (Fig. 3B). Instrument control and quantitation of spectral data were performed using Analyst 1.4.2 and MultiQuant software (AB Sciex Inc.). Intraday coefficient of variation (CV) was determined by analyzing five pooled samples eight times. Intraday CVs for ceramides were C16:0 (3.03%), C18:0(2.49%), C20:0 (5.01%), C22:0 (9.87%), C24:0 (6.92%), C26:0 (8.73%), C16:1 (5.42%), C22:1 (4.78%), C24:1 (4.56%), and C26:1 (4.51%). Intraday CVs for DHCer were C22:0 (7.28%) and C24:0 (7.53%). Interday CVs were determined using five repeat measurements taken over a 3‐month period in each of ten individual plasma samples. Coefficient of variations for ceramide species were C16:0 (3%), C18:0 (14%), C20:0 (13%), C22:0 (10%), C24:0 (11%), C22:1 (14%), C24:1 (13%), and C26:1 (12%), and CVs for DHCer species were C22:0 (13%) and C24:0 (14%). Recovery was determined comparing C12:0 internal standard levels in extracted plasma to equal amounts of C12:0 ceramide prepared in pure methanol and was 90.1%. As the dates of sample collection for this study ranged from 1968 to 2009 and were run in random order over the 3 months, we estimated sample stability by arranging data for each ceramide species by date of study visit. We reasoned that sample degradation or continued enzymatic activity would be manifested by increasing or decreasing trends in ceramide concentrations coincident with storage time. When arranged by date of visit, each species showed a random scatter (Fig. 4), suggesting that ceramide content of plasma was stable with long‐term −80 °C storage.

**Table 5:** Multiple reaction monitoring transitions for molecular species of ceramide molecular and fragment ions

	Molecular/fragment ion *m/z*
Ceramides	
d18:1/12:0	482.9/264.4^a^
d18:1/16:0	538.9/264.4
d18:1/18:0	566.3/264.4
d18:1/20:0	594.8/264.4
d18:1/22:0	622.5/264.4
d18:1/24:0	650.9/264.4
d18:1/26:0	678.9/264.4
d18:1/16:1	536.9/264.4
d18:1/18:1	564.3/264.4
d18:1/20:1	592.8/264.4
d18:s1/22:1	620.5/264.4
d18:1/24:1	648.9/264.4
d18:1/26:1	676.9/264.4
Dihydroceramides	
d18:0/16:0	540.9/266.4
d18:0/18:0	568.3/266.4
d18:0/20:0	596.8/266.4
d18:0/22:0	624.5/266.4
d18:0/24:0	652.9/266.4
d18:0/26:0	680.9/266.4

a Internal standard.

### Statistical analyses

Sex differences in baseline demographic and health‐related characteristics were examined using Fisher's exact test for categorical variables and *t*‐tests or ANOVA for continuous variables. We examined all ceramide species for outliers. As normal ranges for plasma ceramides are not yet known, we conservatively defined an outlier as a concentration of more than three interquartile ranges (25th percentile–75th percentile) from the median; 3.9% of all obtained lipid species were excluded.

Some individuals had missing covariates for a specific visit. Missing values for the covariates described below ranged from 0% to 20.1% (for APOE genotype only) of the 3960 total visits. As the BLSA follows individuals for decades, we imputed the missing covariates for each individual and visit using the nonmissing values of neighboring visits. This allowed us to utilize the largest number of samples.

We used linear mixed models to account for the longitudinal nature of the data and to model the trajectories for individual ceramide class and species over time. Based on the literature, we initially examined the following predictors: age, age squared, sex, race (white vs. African American), education, presence of an APOE E4 allele, BMI, WHR, smoking (never vs. ever), triglycerides, cholesterol, medications (e.g., antidepressants, statins), and medical conditions (e.g., diabetes, hypertension, CKD), and the interaction term between these predictors and age. We forced age, age squared, sex, and BMI into all models and then used backward selection with *P *<* *0.10 to determine which variables to include in the final model for each ceramide and DHCer species. Analyses were conducted using stata version 11.0 (StataCorp LP, College Station, TX, USA). *P *<* *0.05 was used as the threshold for statistical significance.

## Author contributions

MMM conceived the analyses, contributed to the statistical analyses and interpretation of results, and wrote the initial draft of the manuscript. VVRB contributed to the mass spectrometry analyses and interpretation of the data. DH and YA contributed to the statistical analyses, interpretation of results, and writing of the manuscript. RS and LF contributed to the study design of the Baltimore Longitudinal Study on Aging, data collection, interpretation of results and writing of the manuscript. NH contributed to the mass spectrometry analyses, interpretation of results, and writing of the manuscript.

## Funding

This work was supported by a grant from the National Institutes of Health/National Institute on Aging (U01 AG37526) and by the Intramural Research Program of the National Institutes of Health/National Institute on Aging.

## Conflict of interest

The authors have no conflict of interests to declare.
